# Supplemental fibrinogen restores thrombus formation in cardiopulmonary bypass-induced platelet dysfunction *ex vivo*

**DOI:** 10.1016/j.bja.2023.03.010

**Published:** 2023-04-20

**Authors:** Michael Schoerghuber, Thomas Bärnthaler, Florian Prüller, Polina Mantaj, Gerhard Cvirn, Wolfgang Toller, Christoph Klivinyi, Elisabeth Mahla, Akos Heinemann

**Affiliations:** 1 Department of Anesthesiology and Intensive Care Medicine, Medical University of Graz, Graz, Austria; 2 Otto Loewi Research Center, Division of Pharmacology, Medical University of Graz, Graz, Austria; 3 Clinical Institute of Medical Chemical Laboratory Diagnostics, Medical University of Graz, Graz, Austria; 4 Department of Cardiac Surgery, Medical University of Graz, Graz, Austria; 5 Otto Loewi Research Center, Division of Physiological Chemistry, Medical University of Graz, Graz, Austria

**Keywords:** cardiac surgery, cardiopulmonary bypass, coagulopathy, fibrinogen, platelet dysfunction, thrombus

## Abstract

**Background:**

Major cardiac surgery related blood loss is associated with increased postoperative morbidity and mortality. Platelet dysfunction is believed to contribute to post-cardiopulmonary bypass (CPB)-induced microvascular bleeding. We hypothesised that moderately hypothermic CPB induces platelet dysfunction and that supplemental fibrinogen can restore *in vitro* thrombus formation.

**Methods:**

Blood from 18 patients, undergoing first-time elective isolated aortic valve surgery was drawn before CPB, 30 min after initiation of CPB, and after CPB and protamine administration, respectively. Platelet aggregation was quantified by optical aggregometry, platelet activation by flow-cytometric detection of platelet surface expression of P-selectin, annexin V, and activated glycoprotein IIb/IIIa, thrombus formation under flow and effect of supplemental fibrinogen (4 mg ml^−1^) on *in vitro* thrombogenesis.

**Results:**

Post-CPB adenosine–diphosphate and TRAP-6-induced aggregation decreased by 40% and 10% of pre-CPB levels, respectively (*P*<0.0001). Although CPB did not change glycoprotein IIb/IIIa receptor expression, it increased the percentage of unstimulated P-selectin (1.2% *vs* 7%, *P*<0.01) positive cells and annexin V mean fluorescence intensity (15.5 *vs* 17.2, *P*<0.05), but decreased percentage of stimulated P-selectin (52% *vs* 26%, *P*<0.01) positive cells and annexin V mean fluorescence intensity (508 *vs* 325, *P*<0.05). Thrombus area decreased from 6820 before CPB to 5230 after CPB (*P*<0.05, arbitrary units [a.u.]). Supplemental fibrinogen increased thrombus formation to 20 324 and 11 367 a.u. before CPB and after CPB, respectively (*P*<0.001), thereby restoring post-CPB thrombus area to levels comparable with or higher than pre-CPB baseline.

**Conclusions:**

Single valve surgery using moderately hypothermic CPB induces partial platelet dysfunction. Thrombus formation was restored in an experimental study design by *ex vivo* supplementation of fibrinogen.

**Editor's key points**

• Platelet dysfunction contributes to post-cardio-pulmonary bypass (CPB)-induced microvascular bleeding.
• The authors tested whether supplemental fibrinogen can restore *ex vivo* thrombus formation in plasma from patients undergoing aortic valve replacement with moderately hypothermic CPB.
• Aortic valve surgery using moderately hypothermic CPB induced evidence of partial platelet dysfunction that could be corrected by fibrinogen supplementa-tion.
• Clinical studies are needed to define this platelet dysfunction further and to assess the role for fibrinogen as a potential treatment strategy.

Large observational studies have shown an association between the severity of cardiac surgery-related bleeding and 30-day postoperative morbidity and mortality.^1^^,^^2^ The aetiology of cardiac surgery-related bleeding is multifactorial, including concealed surgical bleeding, thrombocytopenia, platelet dysfunction, impaired thrombin formation, hypofibrinogenaemia, and hyperfibrinolysis.^3^, ^4^, ^5^, ^6^, ^7^ In bleeding patients, perioperative transfusion algorithms based on viscoelastic tests and functional platelets using non-empirical cut-offs allow for an early stepwise approach targeted to restore platelets, fibrinogen, and coagulation proteins.^5^ These algorithms have been shown to reduce bleeding and transfusions, and their implementation has been recommended in current guidelines of the Society of Cardiovascular Anesthesiologists and European Association of Cardiothoracic Surgery and Anesthesiology.^8^, ^9^, ^10^

Recommended target plasma fibrinogen levels to minimise bleeding range from 1.5 to 2.0 g L^−1^.^11^ The empirical cut-offs for platelet transfusion are platelet count ≤50×10^9^ L^−1^ or ≤100×10^9^ L^−1^ in cases of severe ongoing bleeding, platelet dysfunction, or both, respectively.^9^ In thrombocytopaenic patients, high endogenous levels of fibrinogen diminish chest tube drainage and transfusion of fresh frozen plasma and platelets by increasing clot firmness.^12^

The combination of cardiopulmonary bypass (CPB)-induced shear stress, release of platelet agonist adenosine diphosphate (ADP) and reduced ADP hydrolysis with hypothermia lead to platelet activation and subsequent dysfunction.^13^, ^14^, ^15^, ^16^, ^17^ The association between the extent of drug-induced platelet inhibition and surgery-related bleeding in patients undergoing cardiac surgery during dual antiplatelet therapy is reasonably well established.^6^^,^^18^^,^^19^ However, the specific alterations of platelet aggregation, platelet activation, and thrombus formation exerted by CPB, and of platelet interactions with fibrinogen, are largely inconclusive and can vary with temperature used during CPB and complexity of surgery.^12^^,^^13^^,^^16^^,^^20^, ^21^, ^22^

We hypothesised that moderately hypothermic CPB induces platelet dysfunction as evidenced by impaired platelet aggregation, platelet activation, and *in vitro* thrombogenesis, which can be improved by supplemental fibrinogen.

## Methods

### Participants

After approval by the Institutional Review Board (EK 33-067 ex 2021), patients presenting for elective cardiac surgery between February and September 2021 were screened for eligibility for this prospective observational *ex vivo* study. Inclusion criteria were: elective first-time isolated aortic valve surgery with or without ongoing aspirin therapy. Exclusion criteria were: ongoing P2Y_12_ receptor inhibitors, oral vitamin K or non-vitamin K antagonist anticoagulants with preoperative discontinuation for less than that recommended in the guidelines, liver cirrhosis, dialysis, and severe anaemia (haemoglobin ≤10 g dl^−1^).^23^ Patients were enrolled after providing written informed consent.

#### Clinical management

The perioperative care of the patients was at the discretion of the attending physicians, who were blinded to measures of platelet aggregation, platelet activation, and *ex vivo* thrombogenesis. Surgery was performed by the cardiac surgeon on duty. According to institutional protocol, anticoagulation for CPB was established by an initial loading dose of 300 U kg^−1^ unfractionated heparin (Gilvasan, Vienna, Austria) to obtain an activated clotting time ≥400 s that was maintained during CPB by supplemental administration. All patients received tranexamic acid (Pfizer, Vienna, Austria, 15 mg kg^−1^ bolus followed by 8 mg kg^−1^ h^−1^ as continuous infusion until the end of CPB).^24^ We used a standard CPB circuit with a centrifugal pump (Medtronic BBAP40/Medtronic Bio-Console 560; Minneapolis, MN, USA) at a mean pressure of 8–11 kPa (60–82 mm Hg). The circuit contained a CAPIOX® Advance Hardshell Venous Reservoir, a hollow fibre membrane oxygenator (Microporous polypropylene/CAPIOX® FX 25 Advance; Terumo GmbH, Eschborn, Germany) with an integrated arterial filter (Polyester screen type, 32 μm), with integrated heat exchanger (stainless steel). Oxygenator and filter were coated (Terumo Xcoating™ surface coating).

The oxygenator was primed with balanced crystalloid fluid 1600 ml and unfractionated heparin 5000 U. During CPB, non-pulsatile flow was maintained at 2.2–2.6 L min^−1^ m^−2^ with a body temperature of 34°C measured in the urinary bladder. On completion of CPB, anticoagulation was reversed by protamine chloride targeted to normalise ACT.^9^^,^^24^ Transfusion triggers were set to a haematocrit of 20% on CPB and 25% thereafter, unless active bleeding or low cardiac output suggested a need to increase. In case of microvascular bleeding additional protamine, coagulation proteins, and platelets were administered according to the institutional algorithm based on thrombelastography with and without heparinase and functional fibrinogen.^8^^,^^9^

### Blood processing

Blood was drawn from an indwelling central venous catheter: (1) after induction of anaesthesia and before CPB to assess platelet aggregation, platelet activation and *in vitro* thrombogenesis, haematocrit, platelet count, fibrinogen, factor XIII, activated partial thromboplastin time (aPTT), prothrombin time (PT), and antithrombin; (2) 30 min after initiation of CPB to assess platelet aggregation and coagulation parameters; (3) after end of CPB and 3–5 min after protamine administration to assess platelet aggregation, platelet activation and *in vitro* thrombogenesis, haematocrit, platelet count, and coagulation parameters. Sodium citrate (3.6%) was used as anticoagulant. Platelet-rich plasma (PRP) was obtained by centrifugation at 110 ***g*** for 10 min for assessment of platelet aggregation using light transmission aggregometry (LTA), and whole blood was assayed for flow cytometry and *in vitro* thrombogenesis.

#### Coagulation and haematology

Coagulation parameters (PT using Thromborel S®, aPTT using Pathromtin® SL, factor XIII using Berichrom® FXIII and fibrinogen using Multifibren® U) were analysed on the Atellica COAG 360 coagulation analysing system™ (Siemens Healthineers, Vienna, Austria).^25^^,^^26^ Haematocrit and platelet count were measured on XN-10 haematology analysers (Sysmex, Vienna, Austria) using system integrated reagents.

#### Platelet aggregation

LTA is considered the gold standard of platelet function testing.^27^^,^^28^ It measures changes in transmission light through a sample of PRP that occurs when platelets change shape and aggregate upon stimulation with an agonist. LTA is not sensitive to changes in platelet count in PRP within the range of 150–60×10^9^ L^−1^.^28^ To obtain broader information about platelet function, ADP, collagen and thrombin receptor-activated peptide 6 (TRAP), acting via different platelet receptors, were used as agonists. LTA was performed on the integrated four-channel optical aggregometry system on the Atellica COAG 360 System (Siemens Healthcare Diagnostics GmbH, Vienna, Austria). After automated pipetting of 140 μl unadjusted PRP into each channel, aggregation was induced with 20 μl of inducing reagent: collagen (type I) (final concentration, 2 μg ml^−1^; Hyphen BioMed, Neuville-sur-Oise, France), adenosine 5′-diphosphate (ADP) (final concentration, 5 μM; Hyphen BioMed, Neuville-sur-Oise, France), or TRAP (final concentration, 50 μM; Bachem Distribution Services GmbH, Weil/Rhein, Germany). Platelet aggregation was recorded over 10 min, and aggregation curves were calculated using a corresponding reference sample of platelet poor plasma.^25^^,^^26^

#### Platelet activation

Platelet activation was quantified by platelet surface activated glycoprotein IIb/IIIa (reported by monoclonal antibody PAC1) and platelet surface expression of P-selectin and phosphatidylserine (measured by binding of annexin V). PRP was diluted in phosphate-buffered saline with Ca^2+^ and Mg^2+^. For P-selectin/annexin V/PAC-1 staining, platelets were activated by A23187, ADP, or collagen, respectively, for 15 min at 37°C in the presence of anti-P-selectin-FITC, annexin V-FITC, or PAC-1-FITC conjugated antibody. For baseline measurements, respective vehicles were used. For final analysis, the stimulant with highest increase over vehicle was chosen (A23187 for P-selectin and annexin V, and ADP for PAC-1). The samples were washed and fixed, and P-selectin upregulation and PAC-1 and annexin V binding were detected using flow cytometry. Data were expressed as mean fluorescence intensity for baseline and % over vehicle for stimulation for PAC-1 and annexin V, and % positive cells for P-selectin.^29^^,^^30^

Unless otherwise stated, all laboratory reagents were supplied by Sigma (Vienna, Austria). The assay buffer used in flow cytometric staining was Dulbecco's modified phosphate-buffered saline (with or without 0.9 mM Ca^2+^ and 0.5 mM Mg^2+^; Invitrogen, Vienna, Austria). Fibrinogen (Haemocomplettan®) was from CSL Behring (Marburg, Germany). ADP and collagen (Chrono-Par®-collagen fibrils [type I]) from equine tendons were supplied by Probe&Go (Osburg, Germany). Reagents were dissolved in water, ethanol, or dimethyl sulphoxide, diluted into assay buffer yielding a final concentration of the solvents <0.1%. P-selectin and PAC1-FITC antibodies were obtained from Becton Dickenson (Vienna, Austria), CD41 antibody from Invitrogen, and Fibrinogen antibody (A0080, also recognises fibrin) was from DAKO/Agilent (Vienna, Austria). Annexin V FITC was from Biolegend. Measurements were recorded on a BD FACSCanto Flow Cytometer and analysed using Flowjo software (BD Biosciences, Franklin Lakes, NJ, USA).

#### In vitro *thrombogenesis*

*In vitro* thrombogenesis was performed as described.^29^ Vena8 Fluoro+ Biochips (Cellix, Dublin, Ireland) were coated (collagen, 200 μg ml^−1^ at 4°C overnight) and then blocked with bovine serum albumin (10 μg ml^−1^) for 30 min at room temperature. Within 1 h after blood was drawn, it was incubated for 15 min in the dark with 3,3-dihexyloxacarbocyanine iodide (1 μM). Subsequently, blood was separated into different reaction tubes and vehicle or fibrinogen (4 mg–1 ml of blood) was added for 20 min, and 2 min after addition of calcium chloride (1 mM final concentration), blood was perfused over the collagen-precoated perfusion channel with a shear rate of 30 dynes m^−2^. Using Hamamatsu ORCA-03G digital camera (Hamamatsu Photonics, Hamamatsu City, Japan) and Cellix VenaFlux software, thrombus formation was recorded for 4 min with a Zeiss Axiovert 40 CFL microscope with Zeiss A-Plan 10×/0.25 Ph1 lens (Zeiss, Oberkochen, Germany). Computerised image analysis was performed using DucoCell software (Cellix), where area covered by the thrombus was calculated. Data are expressed as arbitrary units (a.u.).

#### Staining of thrombi

Staining of thrombi after *in vitro* thrombogenesis was performed as previously described.^29^ In brief, channels were washed with phosphate-buffered saline once to remove unstable thrombi, and antibodies against CD41 and fibrinogen (1:500) were added and incubated for 1 h at room temperature. After an additional washing step, AF488 and AF594 conjugated secondary antibodies were used for visualisation and subsequently replaced with Vectashield mounting medium (Vector Laboratories, Inc., Burlingame, CA, USA). Photomicrographs were obtained with a confocal laser scanning microscope (Zeiss LSM 510 META, Zeiss) using a 40× objective and analysed with ImageJ.^31^

### Statistical analysis

As estimates of study effect were unavailable, a formal power calculation could not be performed. Based on a previous study^29^ and accounting for a dropout rate of 10%, we estimated that 20 patients would be required to demonstrate an effect of CPB on platelet aggregation, platelet activation, and thrombogenesis.

Statistical analysis was performed using GraphPad Prism 9 (GraphPad Software, Inc., San Diego, CA, USA). Normality was assessed by D'Agostino–Pearson omnibus normality test. Comparisons of groups were performed using Wilcoxon signed-rank and Friedman tests. Wilcoxon test was performed using R.Studio 4.2.1,^32^ followed by correction with the Benjamini–Hochberg procedure to control false discovery rate (fdr).^33^ Probability values of *P*<0.05 were considered statistically significant. Data in graphs are shown as box and whisker plots (minimum to maximum, with single data points; the line in the box indicates the median, bounds of the box indicate the inter-quartile range). Data points represent one individual donor/patient, and the same sample was subjected to all conditions shown in one panel.

## Results

Patient characteristics and procedural variables of the 18 patients undergoing aortic valve replacement are presented in Table 1. Six patients were on perioperative aspirin because of prior cerebrovascular events.

**Table 1:** Patient characteristics and procedural variables. Data are presented as mean (standard deviation, or range for age), and percentage (%). *n*, absolute number; U, units.

Characteristic or variable	Value
Age (yr)	64.9 (43–80)
Sex, ***n*** (male/female)	13/5
Body mass index (kg m^−2^)	29.1 (4.7)
Hypertension, ***n*** (%)	10 (56)
Cerebrovascular disease, ***n*** (%)	3 (17)
Diabetes mellitus, ***n*** (%)	0 (0)
Smoking, ***n*** (%)	2 (11)
Atrial fibrillation, ***n*** (%)	4 (22)
Glomerular filtration rate (ml min^−1^)	92.2 (21.4)
Heparin dose (initial; U)	30 722.2 (5645.1)
Protamine dose (mg)	324.9 (75.2)
Cardiopulmonary bypass time (min)	112.9 (35.8)
Aortic cross clamp time (min)	81.4 (26.3)
Transfusion rate (red blood cells; %)	33.3

### Platelet aggregation

**Fig 1:**
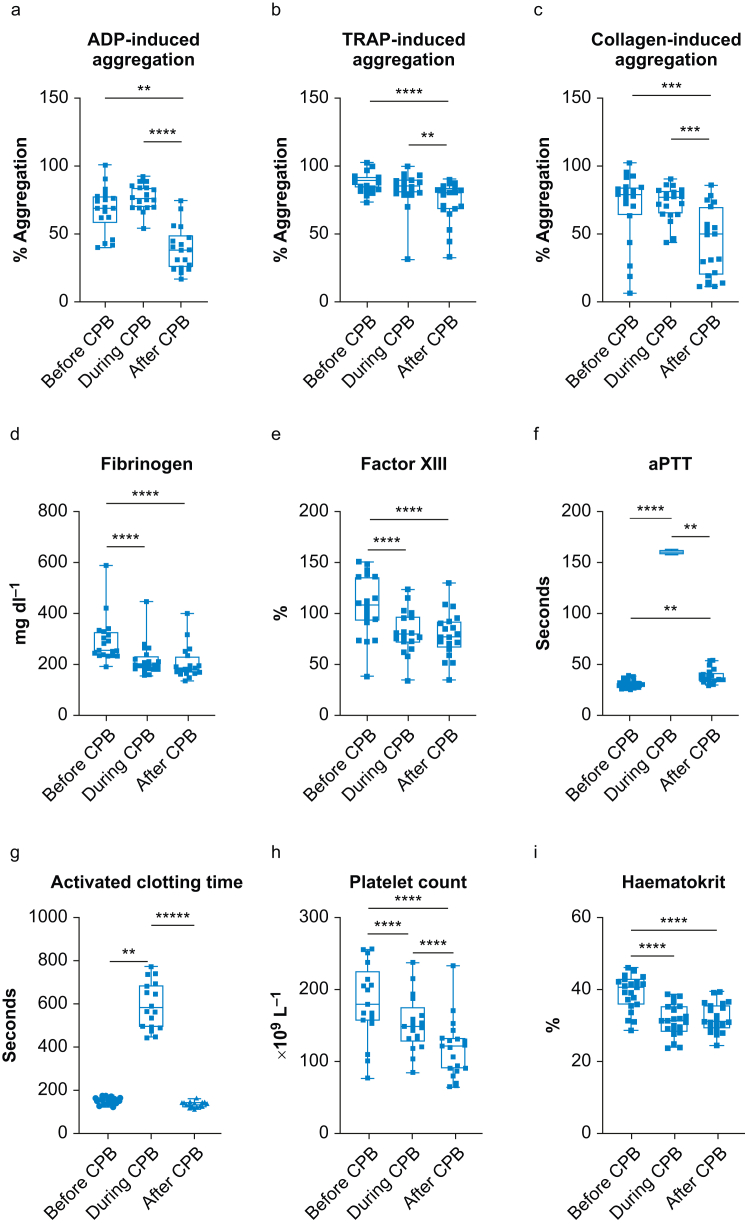
Changes in ADP-, TRAP-, and collagen-induced platelet aggregation, and in fibrinogen and factor XIII levels, activated partial thromboplastin time (aPTT), activated clotting time, platelet count, and haematocrit. Citrated blood was drawn after induction of anaesthesia (before cardiopulmonary bypass [CPB]), 30 min after initiation (during CPB), and after CPB and protamine administration (after CPB). Aggregation in response to (a) 5 μM ADP, (b) 50 µM thrombin receptor activating peptide (TRAP), (c) 2 μg ml^−1^ collagen, (d) fibrinogen level, (e) factor XIII level, (f) aPTT, (g) activated clotting time, (h) platelet count, and (i) haematocrit (HCT). Friedman test followed by Dunn's *post hoc* test was performed to detect differences between groups. A period of 160 s for aPTT is the upper limit. Data are shown as box and whisker plots (minimum to maximum, with single data points; the line in the box indicates the median, bounds of the box indicate inter-quartile range). ∗∗*P*<0.01, ∗∗∗*P*<0.001, ∗∗∗∗*P*<0.0001.

Figure 1 presents ADP-, TRAP-, and collagen-induced platelet aggregation, fibrinogen, factor XIII levels, and aPTT, ACT, platelet count, and haematocrit before CPB, 30 min after initiation of and after termination of CPB and protamine administration. At 30 min after initiation of CPB there was a slight non-significant increase in ADP-induced platelet aggregation. However, after termination of CPB, ADP- and TRAP-induced aggregation decreased by 40% and 10% of pre-CPB levels, respectively (Fig. 1; *P*<0.0001).

Platelet count, fibrinogen and factor XIII were significantly lower after CPB than before CPB, and aPTT returned to pre-CPB levels (Table 2). Haematocrit decreased from 39.2% (35.5–45.5) before CPB to 31.0% (28.8–35.2; *P*<0.0001) thereafter.

**Table 2:** Laboratory measurements. Data are reported as median and inter-quartile range; aPTT, activated partial thromboplastin time; PT, prothrombin time; IQR, inter-quartile range. ∗No valid measurements during cardiopulmonary bypass (CPB), represents upper/lower threshold.

	Before CPB	IQR	During CPB	IQR	After CPB	IQR
aPTT (s)	30.6	4.85	160∗	0	35.3	6
PT (s)	13.4	4.5	90∗	0	19.8	10.8
Fibrinogen (mg dl^−1^)	254	85.5	198	44	180	48
Platelets (×10^9^ L^−1^)	178	55.5	148	32.25	118	47.75
Factor XIIIa (%)	108	39.25	81,5	22.75	79	21
Antithrombin (%)	83	16	70	12	61	13
Haematocrit (%)	39.2	1.22	31.4	1.07	31.0	0.98

### Platelet activation

**Fig 2:**
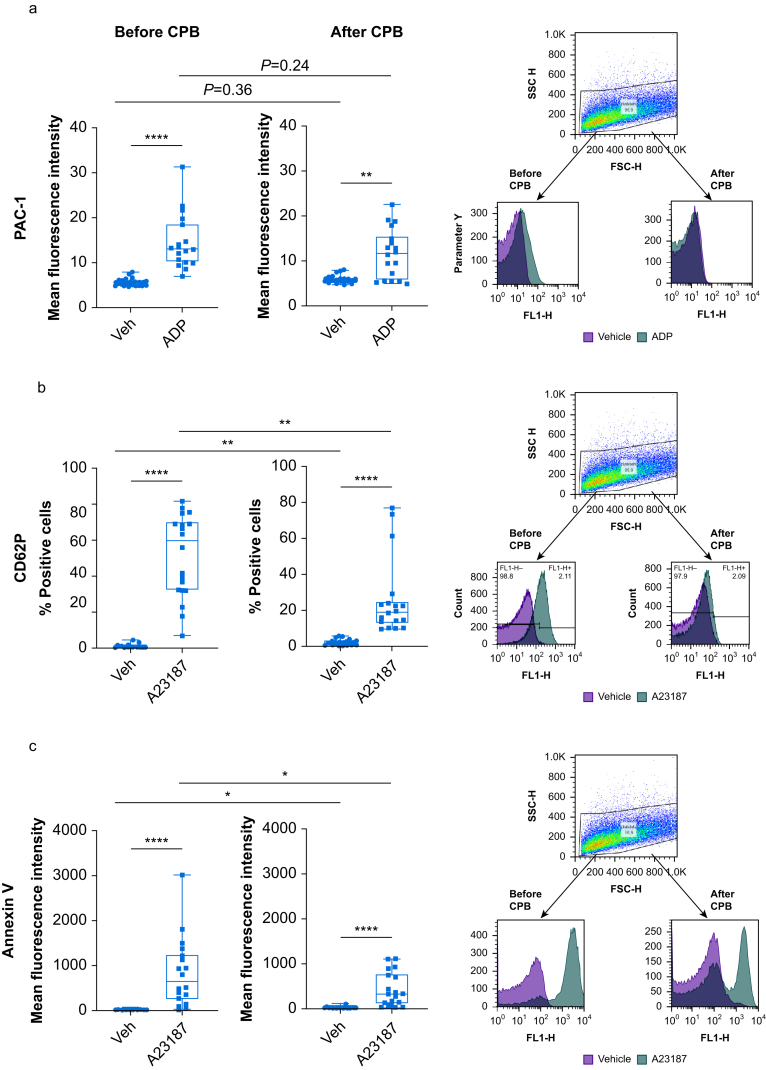
Flow cytometric quantification of platelet surface activated glycoprotein IIb/IIIa and platelet surface expression of P-selectin and phosphatidylserine. Citrated blood was drawn after induction of anaesthesia before (before CPB) and after cardiopulmonary bypass and protamine administration (after CPB). After incubation with vehicle and stimulation with ADP or A23187, (a) PAC-1 expression, mean fluorescence intensity; (b) P-selectin expression, % positive cells; and (c) annexin V expression; mean fluorescence intensity, were measured. Gating strategy and representative histograms for respective staining are shown on the right. Data are shown as box and whisker plots (minimum to maximum, with single data points; the line in the box indicates the median, bounds of the box indicate inter-quartile range). Wilcoxon signed rank test, followed by false discovery rate (fdr) adjustment was performed. ∗*P*<0.05, ∗∗*P*<0.01, ∗∗∗*P*<0.001, ∗∗∗∗*P*<0.0001. veh, vehicle; CPB, cardiopulmonary bypass; ADP, adenosine diphosphate.

Flow cytometric quantification of platelet surface activated glycoprotein IIb/IIIa and surface expression of P-selectin and phosphatidylserine before and after CPB are shown in Figure 2. Flow cytometric staining for PAC1 showed little change induced by CPB with a slight non-significant decrease of ADP stimulated expression after CPB (Fig. 2a). In contrast, after CPB the percentage of unstimulated P-selectin positive platelets significantly increased and A23187-induced P-selectin expression significantly decreased as compared with pre-CPB (Fig. 2b). Similar effects were observed for annexin V, with higher unstimulated values but lower A23791-induced signal after CPB (Fig. 2c).

### *In vitro* thrombogenesis and effect of supplemental fibrinogen

**Fig 3:**
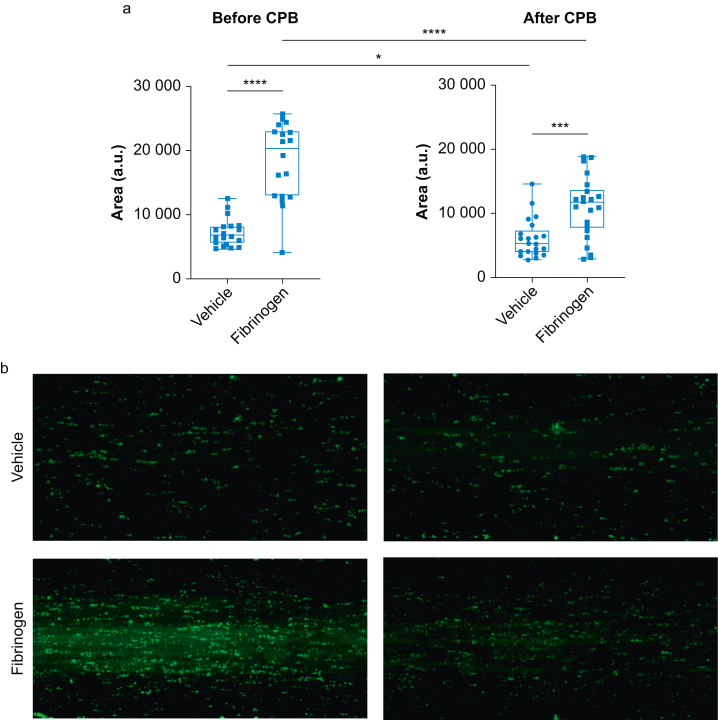
*Ex vivo* thrombogenesis. Whole blood was drawn after induction of anaesthesia before (before CPB) and after cardiopulmonary bypass and protamine administration (after CPB). Blood was incubated with vehicle or supplemental fibrinogen and perfused over collagen precoated channels. (a) The area covered by thrombi is shown as arbitrary units. (b) Respective photomicrographs after 4 min in the absence (upper panel) or the presence (lower panel) of fibrinogen. Data are shown as box and whisker plots (minimum to maximum, with single data points; the line in the box indicates the median, bounds of the box indicate inter-quartile range). Wilcoxon signed rank test, followed by fdr adjustment was performed. *P*<0.05, ∗∗*P*<0.01, ∗∗∗∗*P*<0.0001. CPB, cardiopulmonary bypass; fdr, false discovery rate; a.u., arbitrary units.

Thrombus formation under flow and the effect of supplemental fibrinogen are shown in Figure 3. In order to test whether decreased activation might influence other readouts, we subjected samples to collagen-induced thrombus formation under flow. We found that CPB induced a small but significant decrease in thrombogenesis to 79% of vehicle control (*P*<0.05). Addition of fibrinogen increased thrombus formation under flow both before and after CPB. However, after CPB this increase was only 145% compared with a 245% increase before CPB (*P*<0.001; Fig. 3a). Nevertheless, addition of fibrinogen restored post CPB thrombus formation to levels comparable with or higher than pre-CPB baseline level. Representative photomicrographs, both in the absence and the presence of fibrinogen, are shown in Figure 3b.

### Immunofluorescence analysis of thrombi and the effect of supplemental fibrinogen

**Fig 4:**
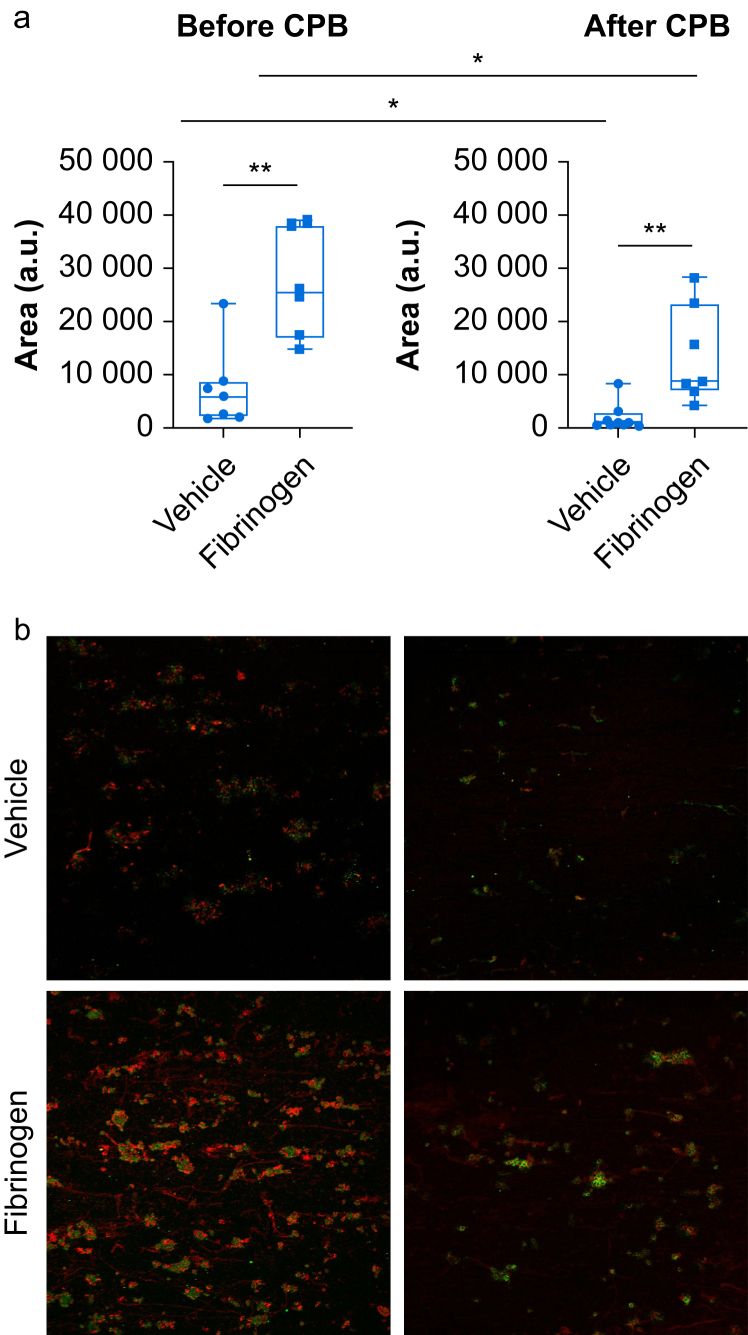
Staining of thrombi after *ex vivo* thrombogenesis. Whole blood was drawn after induction of anaesthesia before (before CPB) and after cardiopulmonary bypass and protamine administration (after CPB). Blood was incubated with vehicle or supplemental fibrinogen and perfused over collagen precoated channels. Thrombi were fixed and stained with antibodies against CD41 (green) and fibrin/fibrinogen (red). (a) Total area covered by fluorescently labelled fibrin(ogen), and (b) representative image showing binding of fluorescently labelled fibrin(ogen) to platelets. Data are shown as box and whisker plots (minimum to maximum, with single data points; the line in the box indicates the median, bounds of the box indicate inter-quartile range). Wilcoxon signed rank test, followed by fdr adjustment was performed to assess significance. ∗*P*<0.05, ∗∗*P*<0.01. CPB, cardiopulmonary bypass; fdr, false discovery rate; a.u., arbitrary units.

Immunolabelling for fibrin/fibrinogen (red) and CD41 (green) is shown in Figure 4 to investigate the composition of the observed thrombi. Supplemental fibrinogen increased the area staining positive for fibrin/fibrinogen before and, to a lesser extent, after CPB (Fig. 4a). Image analysis showed increased fibrin fibre formation after addition of fibrinogen before CPB but very few fibrin fibres after CPB (Fig. 4b).

## Discussion

In patients with microvascular bleeding after CPB, current guidelines support implementation of transfusion algorithms based on predefined transfusion triggers measured by point-of-care devices.^8^^,^^9^ The safety of platelet transfusion is uncertain, and there is no consensus regarding platelet transfusion trigger, dose, and how to measure haemostatic efficacy.^10^^,^^34^^,^^35^

Transient platelet dysfunction after CPB, unrelated to preoperative P2Y_12_ receptor inhibitors, has been described, but is poorly defined and can vary with complexity of surgery. Its impact on cardiac surgery-related bleeding and alternative treatments are largely unknown.^10^^,^^13^^,^^16^^,^^20^, ^21^, ^22^^,^^36^

We prospectively evaluated CPB-induced changes in platelet aggregation, platelet activation, and *in vitro* thrombogenesis with subsequent staining of thrombi with and without supplemental fibrinogen in patients undergoing first-time aortic valve replacement using moderate hypothermic CPB. Acting as a ligand for activated platelet glycoprotein IIb/IIIa receptors, fibrinogen plays an important role in enhancing platelet aggregation and clot formation.^37^

CPB led to a 40% reduction in ADP-induced, but to only a 10% reduction in TRAP-induced, platelet aggregation in patient-derived PRP. Our *ex vivo* data are partially reflected by *ex vivo* data of 490 patients included in the PLATFORM (Prospective LongitudinAl Trial of FFRct: Outcome and Resource IMpacts) study which showed a similar, although more pronounced, post-CPB decrease of the multiple electrode aggregometry ADPtest as compared with the TRAPtest. Importantly, in the PLATFORM study, post-CPB ADPtest achieved the best discrimination of severe bleeding (area under the ROC curve [AUC]=0.712; 95% confidence interval [CI], 0.670–0.752) and the best positive predictive value at an ADPtest ≤ 8 units (PPV=41.7%).^22^ As TRAP is a potent platelet activator via thrombin receptors and at the high concentration used represents the maximum inducible aggregation of platelets, this finding suggests only a slight but significant reduction in post-CPB platelet function.

In contrast to previous work predominantly evaluating alpha granule release, we describe a more comprehensive picture of platelet activation by quantifying platelet surface expression of P-selectin and phosphatidylserine and activated glycoprotein IIb/IIIa receptors.^13^^,^^20^^,^^21^ Fibrinogen cross-links platelets by bridging activated GP IIb/IIIa between adjacent platelets, thereby leading to formation of platelet aggregates.^37^ Upon stimulation, P-selectin is rapidly translocated from platelet alpha granules to the platelet cell surface, and apart from its adhesive properties might have a role in stabilising platelet aggregates.^38^ Plasma coagulation factors assemble on the negatively charged phosphatidylserines exposed on activated platelets, where large amounts of thrombin are generated.^37^

In accordance with previous work, we found increases in P-selectin expressing platelets after CPB.^20^^,^^21^ Although platelets could still be stimulated after CPB using A23718, the magnitude was smaller. Similarly, CPB increased unstimulated, but decreased stimulated, surface expression of annexin V but had no effect on expression of activated glycoprotein IIb/IIIa receptors. As these activation markers were increased after CPB but could not be induced to the same extent by the agonists used, these findings hint towards a complex partial post-CPB platelet defect. Reduced unstimulated and stimulated platelet surface expression of phosphatidylserine as identified by annexin V binding has been associated with both, prior and subsequent bleeding in patients with severe factor VIII deficiency.^30^

CPB induced a small but significant decrease in collagen-induced thrombogenesis under flow. Notably, supplemental fibrinogen restored post-CPB thrombus formation *in vitro* to levels comparable with or higher than pre-CPB baseline. Post-CPB fibrinogen levels were above target plasma fibrinogen levels in treatment guidelines for all patients.^11^

In a previous work, supplemental fibrinogen restored thrombus formation in patients on dual antiplatelet therapy after percutaneous coronary intervention independently of its conversion to fibrin.^29^ In the current investigation, we also found that the area staining positive for fibrin/fibrinogen was increased after fibrinogen addition both before and, to a lesser extent, after CPB. There were more fibrin fibres before CPB irrespective of supplementation. The fact that these fibres were rarely observed after CPB but supplemental fibrinogen still resulted in an overall increase in thrombus formation suggests crosslinking of platelets as the predominant mechanism of fibrinogen in collagen-dependent thrombus formation under flow. Although thrombus formation after CPB was restored by fibrinogen, the magnitude was slightly lower compared with fibrinogen treatment before CPB.

In dilutional coagulopathy, fibrinogen restores the rarefied fibrin meshwork induced by dilution and dose-dependently increases clot firmness, similar to or even more than platelet transfusion in thrombocytopaenic patients.^39^, ^40^, ^41^ In thrombocytopaenic patients after CPB, endogenous fibrinogen levels >240 mg dl^−1^ restore clot firmness and decrease overall chest tube drainage.^12^ The current investigation corroborates and extends the above findings by demonstrating *in vitro* efficacy of supplemental fibrinogen in partially dysfunctional platelets using a microchip-based flow chamber whole blood assay that more reliably mirrors the dynamic interactions of erythrocytes, platelets, and coagulation factors compared with platelet reactivity measures under static conditions.^42^

In patients undergoing complex cardiac surgery, three RCTs have evaluated fibrinogen supplementation, targeted to ROTEM FIBTEM based maximal clot strength of 22 mm or to a plasma fibrinogen level of 2.5 g L^−1^ compared with placebo. In the REPLACE trial, more units of allogenic blood components were administered during the first 24 h after fibrinogen administration compared with placebo.^43^ In contrast, reduced cumulative 24-h postoperative chest tube drainage and a greater avoidance of any allogenic blood products in fibrinogen-treated patients as compared with placebo, respectively, had also been reported.^44^^,^^45^ This discrepancy highlights the need for further studies.

There are several important limitations to the current study. All patients underwent first-time elective aortic valve replacement using moderately hypothermic CPB. The observed post-CPB platelet dysfunction in these patients was mild, which might be worse after complex cardiac surgery and deep hypothermia.^16^ In contrast to previous studies, we only investigated post-CPB platelet aggregation, activation, and *ex vivo* thrombus formation in comparison with pre-CPB baseline.^13^^,^^20^^,^^21^ As previous papers demonstrated the transient nature of CPB-induced platelet dysfunction with recovery at 24 h after CPB, we believe that these timepoints are substantiated by current guidelines advocating early and targeted coagulation management in microvascular bleeding after CPB.^6^^,^^9^ Although we show that fibrinogen supplementation restores post-CPB thrombus formation to levels above pre-CPB baseline, our experimental data cannot yet guide clinical decision making. Clinical data are needed in post-CPB patients with microvascular bleeding to support or refute efficacy of supplemental fibrinogen as a potential alternative treatment in the context of platelet dysfunction.^10^ Furthermore, the experimental design of our investigations precludes us from judging with certainty whether fibrinogen improves platelet dysfunction *per se* or ameliorates thrombus formation via increased crosslinking. However, our previous study showing amelioration of thrombus formation after dual antiplatelet therapy by fibrinogen and the fact that thrombus formation under flow is slightly lower after CPB makes the second explanation more likely.^29^

In conclusion, aortic valve surgery using moderately hypothermic CPB induces *ex vivo* evidence of partial platelet dysfunction that can be corrected by supplementation of fibrinogen in the flow chamber. Clinical studies are needed to delineate platelet dysfunction further, particularly in complex cardiac surgery, and to assess a potential role for fibrinogen as an alternative treatment strategy.

## Authors’ contributions

Experiments: TB, MS, FP, GC, PM

Project conception: AH, EM, WT, FP, PM, MS, TB

Study design: AH, EM, WT, FP, TB

Data collection: MS, FP, GC, CK, PM and PM

Data analysis: TB, MS, GC, CK, FP, EM

Preparation of figures: TB

Writing of article: TB, MS, AH, GC, WT, EM

Final approval of the article: TB, MS, FP, PM, GC, WT, CK, EM, AH

## Acknowledgements

We acknowledge Ilse Lanz, Eva Tatzl, and the laboratory technicians of the central laboratory for their excellent technical assistance.

## Declaration of interest

The authors have no conflicts of interest to declare.

## Funding

Departmental resources. TB is a recipient of the Apart-Mint (ÖAW, 11974) and Schrödinger Fellowship (FWF, J-4547).
